# Post-transcriptional regulation of 5'-untranslated regions of human Transient Receptor Potential Vanilloid type-1 (TRPV-1) channels: role in the survival of glioma patients

**DOI:** 10.18632/oncotarget.13132

**Published:** 2016-11-05

**Authors:** Massimo Nabissi, Maria Beatrice Morelli, Antonietta Arcella, Claudio Cardinali, Matteo Santoni, Giovanni Bernardini, Angela Santoni, Giorgio Santoni, Consuelo Amantini

**Affiliations:** ^1^ School of Pharmacy, Experimental Medicine Section, University of Camerino, Camerino (MC), Italy; ^2^ Department of Molecular Medicine, Sapienza University, Rome (RM), Italy; ^3^ I.N.M. Neuromed, Pozzilli, Isernia (IS), Italy; ^4^ Department of Medical Oncology, AOU Ospedali Riuniti, Polytechnic University of the Marche Region, Ancona (AN), Italy; ^5^ School of Biosciences and Veterinary Medicine, University of Camerino, Camerino (MC), Italy

**Keywords:** TRPV1, glioblastoma, 5'UTR, survival rate, prognosis

## Abstract

The Transient Receptor Potential Vanilloid type-1 (TRPV1) channel is a non-selective cation channel belonging to the Transient Receptor Potential family; variation of its expression has been correlated to glioma progression. In human, TRPV1 transcripts display a remarkable homogeneity differing only for the 5'-untranslated region (5'UTR) sequence that generates four variants encoding the same protein. Herein, we investigated the role of the 5'UTR sequences in TRPV1 transcripts stability, regulation of translation, expression in glioma cells and tissues. In addition, the expression of 5'UTR TRPV1 variants as prognostic factor in the survival of glioblastoma patients was evaluated. The expression level for each 5'UTR and their stability was evaluated by RT-PCR analysis. The effect of rapamycin and interferon-gamma in 5'UTR-regulating TRPV1 translation was determined by western blot analysis in glioma cell lines. We demonstrated that the 5'UTR influences the stability and translation efficacy of TRPV1 transcripts, and that TRPV1 variant three (TRPV1_v3_) was the most stable and the only variant expressed in GBM samples and in glioma stem-like cells. Furthermore, we found that TRPV1_v3_ expression levels correlate with patient's survival, suggesting that it may represent a potential prognostic marker for patients with glioma.

## INTRODUCTION

The Transient Receptor Potential Vanilloid type-1 (TRPV1) channel is a non-selective cation channel structurally related to members of the Transient Receptor Potential (TRP) family. The gene encoding the human TRPV1 (ENSG00000196689) is located on Chromosome 17: 3,565,444-3,609,411 reverse strand and covers a region of 44 Kb. The canonical form of TRPV1 comprises 839 aa (MW ≈ 96 kDa) and is composed of six trans-membrane spanning domains and a pore loop domain between trans-membrane domains 5 and 6 [1]. The N-terminal and C-terminal tails are on the cytoplasmic side. Three N-terminal ankyrin (ANK) repeats spanning amino acids 101-304 are present in N-terminal tail [2]. In TRP channel families, alternative splicing enables the same gene to generate multiple mature RNA types for translation, resulting in multiple channel proteins [3]. At present three different TRPV1 splice variants have been identified: the Vanilloid Receptor 5’ splice variant (VR.5'sc), the TRPV1beta (TRPV1β) and the TRPV1variant (TRPV1var) [4]. The VR.5'sc variant, identified in rat, originates from an alternative initiation of translation resulting in a truncated N-terminal intracellular domain and on the skipping of an exon, resulting in the loss of 60 aa encoding for a N-terminal intra-terminal region including the third ankyrin repeat domain; the TRPV1β, described in a human clone that lacked exon 7 (30 nucleotide loss) encoding a portion of the third ankyrin repeat domain [5]; TRPV1var isolated from rat kidney papilla resulting from the failure to splice out intron 5 [6]. In addition, although TRPV1 transcripts display a remarkable homogeneity in the open reading frame and in the 3’-untranslated regions (3'UTR), different 5'-untranslated region (5'UTR) sequence due to alternative 1st exon, generates four variants (TRPV1_v1_, TRPV1_v2_, TRPV1_v3_, TRPV1_v4_) encoding the same protein, with TRPV1_v2_ sharing 100% homology with TRPV1 wild type (TRPV1_v0_). In-frame stop codon immediately upstream of the translation start site ensures that the 5’ variability occurs at the mRNA level, with only the 5'UTR affected by the alternative 1st exon [2]. The 5'UTR is a major site of regulation of translation, since it contains both *cis*- and *trans*-acting elements that contribute to qualitative and quantitative regulation of this process [7]. Elements in 5'UTR, such as upstream open reading frames (uORFs), internal ribosome entry sites (IRES) [8, 9], RNA G-quadruplexes [10], terminal oligopyrimidine tract (TOP) [11], gamma interferon activated inhibitor of ceruloplasmin mRNA translation (GAIT) [12], have been demonstrated to affect translation. In addition, the length and the GC content in the 5'UTR can influence the translation of the main ORFs [13, 14]. So, 5'UTR elements can influence regulation of gene expression and are associated with the development of human diseases by controlling mRNA stability and translational efficiency, as in cancer [15]. In human cancer, TRPV1 has been found to activate a cell death program in prostate [16], colon [17], pancreas [18], breast [19] and bladder cancer [20, 21]. Moreover, TRPV1 is also involved in glioma progression and changes of its expression contribute to malignancy. We have previously reported that TRPV1 is expressed in glioma tissues but not in epileptic brain tissues, with an inverse correlation between TRPV1 expression levels and pathological glioma grade [22]. Moreover, neural precursor cell stimulation of TRPV1 has been found to trigger high-grade glioma cell death, activating the ATF3 transcription factor controlling ER stress pathway [23, 24]. Finally, it has been recently reported at mRNA level, that TRPV1 was over-expressed in glioblastoma (GBM) patients with higher overall survival (OS, more than 12 months) as respect to those showing lower OS (less than 12 months) [25]. Since 5'UTRs of the human TRPV1 transcripts could play a role in TRPV1 stability and translational efficiency, we investigated the involvement of 5'UTR sequences in TRPV1 stability, regulation of TRPV1 protein synthesis and expression in GBM cell lines. Moreover, the expression of 5'UTR TRPV1 variants in GBM, glioma stem-like cells (GSCs) and the 5'UTR TRPV1 variants role as prognostic factor in the survival of GBM patients have been evaluated.

## RESULTS

### The 5'UTR regions influence the RNA secondary structure of TRPV1 transcripts

**Figure 1:**
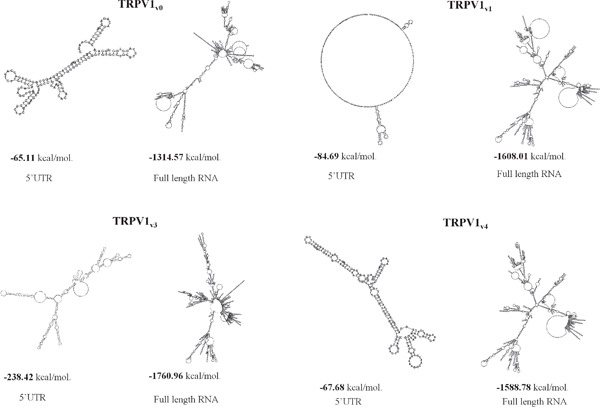
Secondary structure and free-energy value of the TRPV1 5'UTR variants and full-length sequences Folding of 5'UTR TRPV1 variants and of the corresponding full length TRPV1 RNA transcripts predicted by the mFold program. Predicted secondary structures of lowest free energy were represented in kcal/mol.

The 5'UTR of TRPV1 mRNA transcripts were selected from the mRNA sequences available in UNIGENE database. Five 5'UTR variants, named TRPV1_v0-4_, were present and they differed for the length of 5'UTR (Table 1), except for TRPV1_vo_ and TRPV1_v2_ that showed 100% nucleotide sequence homology. ORF and 3'UTR regions, showed 100% nucleotide sequence homology in all the TRPV1 5'UTR variants. Therefore, for the structure analysis of TRPV1 5'UTR we selected TRPV1_v0_, _v1_, _v3_, _v4_ variants. First, the evaluation of the 5'UTR length and GC content evidenced that the TRPV1_v3_ is the longest (527 nt), followed by TRPV1_v1_ (275 nt), TRPV1_v4_ (201 nt) and TRPV1_v0_ (158 nt), while GC content was higher in TRPV1_v0_ (65.8%), followed by TRPV_v3_ (62.2%), TRPV1_v1_ (58.2%) and TRPV1_v4_ (54.7%) (Supplementary Table 1). Since the 5'UTR sequence appeared to influence the overall topology of the complete mRNA and the GC content was correlated with the thermodynamic stability of RNA secondary structure, we predicted the free energy of the secondary structure both of 5'UTRs and of the full length TRPV1 5'UTR variant sequences, using the mFold software. As shown (Figure 1) the TRPV1_v3_ transcript has the lower free energy, followed by TRPV_v4_, TRPV1_v1_ and TRPV_v0_, while considering the 5'UTR, TRPV1_v3_ lower free energy was followed by that of TRPV1_v1_, TRPV1_v4_ and TRPV1_v0_. Overall, for any TRPV1 variant, we evaluated the RNA sequence length, the GC content and the estimated free energy. Since the mRNA with the most negative value of free energy is considered the most structured and the most stable, these results suggest that TRPV1_v3_ should be the most stable TRPV1 variant transcript.

**Table 1:** Comparative sequence analysis of the TRPV1 5'UTR variants

TRPV1_v3_ |ref|NM_080706.3|	CTGCCGCTCACCCTATTCCAGGGACA-CAGTCTGCTTGGCTCTTCTGGAC	49
TRPV1_v4_ |ref|NM_080705.3|	-----------------------GCA-GAGTGTGC--AG----TATAGAT	20
TRPV1_v1_ |ref|NM_080704.3|	----------------------GGCTCAGGCAGGCCTGG----CCCAGAG	24
TRPV1_v0_ |ref|NM_018727.5|	-----------------------GCCCGGG--------A----CCC----	11
gi|117306163|ref|NM_080706.3|	TGAGCCATCCTCATCACCGAGATCCTCCCTGAATTCAGCCCACGACAGCC	99
gi|117306162|ref|NM_080705.3|	TCAGCGTTTGTCG--ACTGA--------CTGAATG------ATAGCA-CA	53
gi|117306161|ref|NM_080704.3|	TCA-CGCTGGCAACCAC-GAG------TTTGGGAA------GCAGTCGTA	60
gi|117306160|ref|NM_018727.5|	-CA-CGGAGGCG------------------GGGAG------AC-------	28
gi|117306163|ref|NM_080706.3|	ACCCCGGCCGTTTTCCTTGTTCTGTGTGGGGAGGGAGGCAGCGCGGTGGT	149
gi|117306162|ref|NM_080705.3|	ATCCCGGCTGCTTCC--TGTT-----------------------------	72
gi|117306161|ref|NM_080704.3|	TTCTCTCTCTCTCTCTCTCTC-----------------------------	81
gi|117306160|ref|NM_018727.5|	----CACTCT----------------------------------------	34
gi|117306163|ref|NM_080706.3|	TATCAACCTCACCCTGCAGAGGAGGCACCTGAGGCCCAGAGACGAGGAGG	199
gi|117306162|ref|NM_080705.3|	-------------------------------------------------G	73
gi|117306161|ref|NM_080704.3|	----------------------------------------------TCTC	85
gi|117306160|ref|NM_018727.5|	----------------------------------------------TCTC	38
gi|117306163|ref|NM_080706.3|	GATGGGTCTAACCCAGAACCACAGATGGC-TCTGAGCCGGGGGCCTGTCC	248
gi|117306162|ref|NM_080705.3|	GCTGGGTTT-----------------GGT-T-------------------	86
gi|117306161|ref|NM_080704.3|	TCTCAGTATCCATGA----------CAGTGTGAT----------------	109
gi|117306160|ref|NM_018727.5|	CCACACGAGCC--------------CAG----------------------	52
gi|117306163|ref|NM_080706.3|	ACCCTCCCAGGCCGACGTCAGTGGCCGCAGGA------CTGCCTGGGCCC	292
gi|117306162|ref|NM_080705.3|	-----------------------------GGA------CTG---GGACCC	98
gi|117306161|ref|NM_080704.3|	-----------------------------GGAGAGTCTCTGCC-GTGCC-	128
gi|117306160|ref|NM_018727.5|	------------------------------------CTCTCCC-------	59
gi|117306163|ref|NM_080706.3|	TGCTAGGCCTGCTCACCTCTGAGGCCTCTGGGGTGAGAGGTTCAGTCCTG	342
gi|117306162|ref|NM_080705.3|	--------------------------------GTCAGAGG----------	106
gi|117306161|ref|NM_080704.3|	--------------------------------ATCTGGGATGC-------	139
gi|117306160|ref|NM_018727.5|	---------------------------------TTCGAGTAGC-------	69
gi|117306163|ref|NM_080706.3|	GAAACACTTCAGTTCTAGGGGGCTGGGGGC----AGCAGCAAGTTGGAGT	388
gi|117306162|ref|NM_080705.3|	AAAA-----------------------GGC----AAC-------------	116
gi|117306161|ref|NM_080704.3|	AAACCGTCCCTGTGTTCCCCACGTCCAGGCCGTAGAT-------------	176
gi|117306160|ref|NM_018727.5|	AA----------------CCGCCTTCAAGC--------------------	83
gi|117306163|ref|NM_080706.3|	TTTGGGGTACCCTGCTTCACAGGGC----CCTTGGCAAGGAGGGCAGGTG	434
gi|117306162|ref|NM_080705.3|	-----------------------GC----CGCTGACAAAGAACATTGCCG	139
gi|117306161|ref|NM_080704.3|	-----------------------GCTCCCCGCCGGTCAGTCACTTAGTCG	203
gi|117306160|ref|NM_018727.5|	---------------------------------------TCAC-------	87
gi|117306163|ref|NM_080706.3|	GGGTCTAAGGACAAGCAGTCCTTAC---TTTGGGAGT---CAACCCCGGC	478
gi|117306162|ref|NM_080705.3|	A-----AAGG----------TTCA------TGGGAG------GCTCCGGC	162
gi|117306161|ref|NM_080704.3|	T-----CAGATCG-------CCCGT---CCTGGTATCACAGTGCTTCTGT	238
gi|117306160|ref|NM_018727.5|	--------AAGCA-------CCCGTGGGCCTGGGGT----GTGCCTGCGT	118
gi|117306163|ref|NM_080706.3|	GTGGTGGCTGCTGCAG---GTTGCACACTGGGCCACAGAGGATCCAGCAA	525
gi|117306162|ref|NM_080705.3|	----------TAACAG---GTTGCACACTGGGCCACAGAGGATCCAGCAA	199
gi|117306161|ref|NM_080704.3|	------------TCAG---GTTGCACACTGGGCCACAGAGGATCCAGCAA	273
gi|117306160|ref|NM_018727.5|	------------CTAGCTGGTTGCACACTGGGCCACAGAGGATCCAGCAA	156
gi|117306163|ref|NM_080706.3|	GGATGAAGAAATGGAGCAGCACAGACTTGGGGGCAGCTGCGGACCCACTC	575
gi|117306162|ref|NM_080705.3|	GGATGAAGAAATGGAGCAGCACAGACTTGGGGGCAGCTGCGGACCCACTC	249
gi|117306161|ref|NM_080704.3|	GGATGAAGAAATGGAGCAGCACAGACTTGGGGGCAGCTGCGGACCCACTC	323
gi|117306160|ref|NM_018727.5|	GGATGAAGAAATGGAGCAGCACAGACTTGGGGGCAGCTGCGGACCCACTC	206

Gene bank accession number (gi and NM_) are indicated.

### The 5'UTR modulates the half-life of TRPV1 variant transcripts

**Figure 2:**
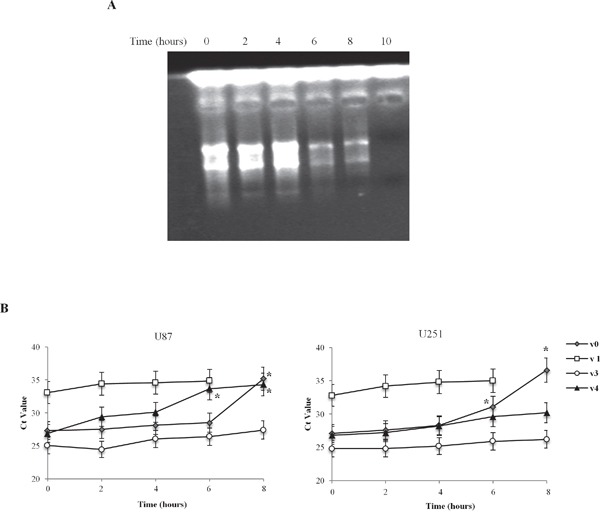
Time-dependent TRPV1 5'UTR variant transcripts stability in U87 and U251 cell lines **A.** Representative image of total RNA from Act D-treated U87 and U251 glioma cell lines analyzed by agarose gel electhrophoresis, up to 10 h post-treatment, **B.** Ct value at different times (2, 4, 6 and 8 h) post-Act D treatment. Time 10 h was omitted since no TRPV1 transcripts expression was detected. Data were obtained by RT-PCR analysis and represented one out of three separate experiments.

In order to investigate the role of the 5'UTR in regulating mRNA stability, we measured the half-life of the TRPV1 5'UTR variant mRNAs, in U87 and U251 glioma cell lines. The two cell lines were treated with actinomycin D (Act D, 10 μM) to block transcription, and the exponential decay was followed up to 10 h post-treatment. As shown (Figure 2A), the effect of Act D on RNA transcription, evaluated by agarose gel electrophoresis, clearly demonstrated a reduction of total RNA quantity, just after 6 h of treatment. The results indicate that the TRPV1 variants are expressed at different levels in both cell lines (Figure 2B). Higher TRPV1_v3_ level, followed by TRPV1_v4_, TRPV1_v0_ and TRPV_v1_ were observed. Time course analysis in Act D-treated samples evidences that the TRPV1_v3_ is the most stable with a Ct decay (Ct time 0 – Ct time 8, Δ Ctd) of 2, followed by TRPV1_v4_ (ΔCtd = 7.4), TRPV1_v0_ (ΔCtd = 8.3) and finally TRPV1_v1_ with a total decay, after 8 h of treatment. Overall, these results indicate that TRPV1 variants stability is correlated to their free energy value, confirming that TRPV1_v3_ is the most stable transcript.

### TOP motif in 5'UTR regulates TRPV1 translation

**Figure 3:**
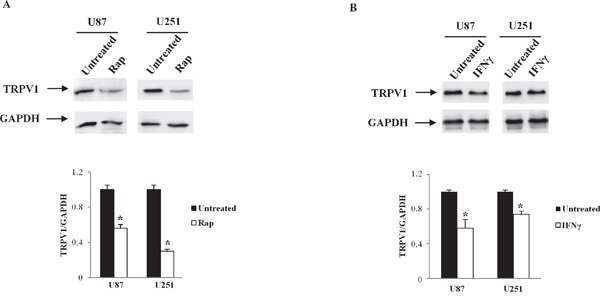
TRPV1 translation regulation by Rap and IFNγ in glioma cell lines U87 and U251 glioma cell lines were treated with Rap (10 μM), or IFNγ (100 μM) and cellular lysates from treated glioma cells were immunoblotted with anti-TRPV1 antibody. GAPDH was used as loading control. Densitometric analysis was performed by using a ChemiDoc apparatus and software. Data presented are one out of three separate experiments.

Since motifs present in 5'UTR are known to regulate protein translation, we analyzed TRPV1 5'UTR sequences by using RegRNA, a regulatory RNA motifs and element finder. As shown (Table 2), software analysis identified different regulatory elements in all of the TRPV1 5'UTR sequences. uORF motif was found in each TRPV1_v1, v3, v4_ and an IRES was found in TRPV1_v4_ only. Regarding uORF, it is considered that all type of uORF can reduce protein expression, and specifically, three uORF properties are associated with greater inhibition: strong uAUG context, increased distance from the cap (cap-uORF distance > 50 nt negative influence translation) and multiple uORF in the 5'UTR. Sequence analysis of TRPV1 5'UTRs indicated similar properties in all TRPV1 5'UTR sequences: one single uORF is present in TRPV1_v1,3,4_, cap-uORF > 50 and strong uAUG context. These analyses suggest that uORF in TRPV1 5'UTR should not influence different translation efficacy in TRPV1 5'UTR variants. TOP that makes mRNA translation sensitive to rapamycin (Rap) was found in all TRPV1 5'UTRs sequences. To address the importance of the 5'TOP motif in serving as inhibitory target of Rap in suppressing the translation of the TRPV1 5'UTR variants, we treated U87 and U251 cell lines with 10 μM Rap for 24 h. TRPV1 protein levels, evaluated by western blot analysis, in Rap-treated cells, evidenced a strong reduction of TRPV1 translation compared with control cells (Figure 3A), suggesting that Rap, through 5'TOP motif, was able to suppress TRPV1 translation. In addition, by RegRNA analysis, the presence of an interferon-gamma (IFN-γ) activated inhibitor of translation (GAIT) element was determined in 5’ UTR of TRPV1_v0, v3, v4_. Since mRNAs with GAIT element are supposed to be negatively regulated by IFN-γ, we treated U87 and U251 cell lines with IFN-γ (100 μM) for 24 h. After treatments, we analyzed the levels of TRPV1 transcript by western blot analysis, and the results evidenced a reduced expression of TRPV1 proteins in both glioma cell lines, mainly in U87 cells (Figure 3B), suggesting that IFN-γ was able to negatively regulate GAIT-TRPV1 variants translation, with levels of TRPV1 protein likely sustained by the TRPV_v1_ expression that does not contain a GAIT element in its 5'UTR region.

**Table 2:** Motifs in TRPV1 5'UTR

TRPV1_v0_				
No.	RegRNA ID	Location	Length (nt)	Sequence
1	TOP	53-63	11	CTCTCCCTTCG
2	GAIT	74-98	25	CGGTTGCTACTGAAGGGAGAGCTG
		107-134	28	GTGTGCCTGCGTCAGCTGGTTGCACAC

TRPV1_v1_				
No.	RegRNA ID	Location	Length (nt)	Sequence
1	TOP	117~121	5	CTCTG
		147~151	5	CCCTG
		178~184	7	CTCCCCG
		232~237	6	CTTCTG
2	uORF	97~174	78	ATGACAGTGTGATGGAGAGTCTCTGCCGTGC
				CATCTGGGATGCAAACCGTCCCTGTGT
				TCCCCACGTCCAGGCCGTAG

TRPV1_v3_				
No.	RegRNA ID	Location	Length (nt)	Sequence
1	TOP	39~46	8	CTCTTCTG
		73~80	8	CCTCCCTG
		101-105	5	CCCCG
		114-118	5	CCTTG
		161-165	5	CCCTG
		228-232	5	CTCTG
		290-294	5	CCCTG
		308-313	6	CCTCTG
		317-322	6	CCTCTG
		398-402	5	CCCTG
		413-418	6	CCCTTG
		459-463	5	CTTTG
		472-476	5	CCCCG
2	uORF	201-314	114	ATGGGTCTAACCCAGAACCACAGATGGCTC
				TGAGCCGGGGGCCTGTCCACCCTCCCAGGC
				CGACGTCAGTGGCCGCAGGACTGCCTGGGC
				CCTGCTAGGCCTGC**TCACC**TCTGA
3	GAIT	27-51	25	CAGTCTGCTTGGCTCTTCTGGACTG
		110-134	25	TTTTCCTTGTTCTGTGTGGGGAGGG
		110-136	27	TTTTCCTTGTTCTGTGTGGGGAGGGAG
		110-137	28	TTTTCCTTGTTCTGTGTGGGGAGGGAGG
		392-416	25	GGGGTACCCTGCTTCACAGGGCCCT
		438-465	28	TCTAAGGACAAGCAGTCCTTACTTTGGG
		109-133	25	CGGCCGGGGTGGCTGTCGTGGGCTG

TRPV1_v4_				
No.	RegRNA ID	Location	Length (nt)	Sequence
1	TOP	64-70	7	CTTCCTG
		156-160	5	CTCCG
2	uORF	43-165	123	ATGATAGCACAATCCCGGCTGCTTCCTGTT
				GGCTGGGTTTGGTTGGACTGGGACCCGTCA
				GAGGAAAAGGCAACGCCGCTGACAAAGAAC
				ATTGCCGAAAGGTTCATGGGAGGCTCCGGCTAA
4	GAIT	19-44	26	ATTCAGCGTTTGTCGACTGACTGAAT

RegRNA ID are represented, Location (as nucleotide position in 5'UTR mRNA region, Length in bases (b) and the relative nucleotide sequence. Terminal Oligopyrimidine Tract (TOP), Gamma Interferon activated inhibitor of Ceruloplasmin mRNA translation (GAIT), Upstream Open Reading Frame (uORF), Internal Ribosome Entry Site (IRES).

### TRPV1 5'UTR variants expression in glioma tissues and GSCs

To evaluate which TRPV1 5'UTR variant was expressed in glioma samples, we analyzed the expression of TRPV1 5'UTR variants in different glioma biopsies, from grade I to IV (n = 84) obtained from mixed patients (Supplementary Table 2), in normal brain (NB; n= 3) and in normal human astrocytes (NHA; n = 3) samples, by RT/PCR analysis. As shown NB, NHA and glioma grade I samples expressed at different levels all the 5'UTR TRPV1 variant transcripts, while some samples resulted express the TRPV1 variants: grade II (10/12), III (12/20) and IV samples (38/40) (Table 3A). Furthermore, we found that the TRPV1 variants expression levels strongly decreased in TRPV1 positive (TRPV1^+^) glioma samples (grade III and IV) (Table 3A), except for the TRPV1_v3_, which expression was maintained in grade IV TRPV1^+^ samples (Table 3A). In fact, the TRPV1_v3_ was found to be the only TRPV1 5'UTR transcript expressed in the majority (n = 38/44) of GBM samples (Table 3A). Moreover, we found that the GBM samples showed different TRPV1_v3_ relative expression (Rel. expr.) level, with 50% of GBM samples showing low expression (Rel. expr. ≤ 0.5), 36.4 % with high expression (Rel expr. > 0.5) and 13.6 % with complete loss of TRPV1_v3_ expression (Rel. expr. 0) (Table 3B). Evaluation of TRPV1 variant transcripts was also performed in Human Normal Progenitor Cells (HNPCs; n = 3), in Glioma Stem-like Cells (GSCs; n = 4) and in differentiated GSCs (dGSCs; n = 4). As shown (Table 3C), HNPCs sample expressed all the TRPV1 variants, with similar levels found in NHA, two of four GSCs were shown to express TRPV1_v3_ only, while after differentiation all the GSCs showed expression of all the TRPV1 variants (Table 3C).

**Table 3:** TRPV1_v3_ expression in GBM samples and GSCs

A)						
TRPV1 5'UTR variants	NB (n=3/3)	NHA (n=2/2)	Grade I (n=8/8)	Grade II (n=10/12)	Grade III (n=12/20)	Grade IV (n=38/44)
TRPV1_v0_	0.42 ± 0.4	0.22 ± 0.01	0.62 ± 0.22	0.34 ± 0.01	0.16 ± 0.01	0
TRPV1_v1_	0.61 ± 0.09	0.72 ± 0.11	0.33 ± 0.12	0.26 ± 0.01	0.03 ± 0.01	0
TRPV1_v3_	0.85 ± 0.12	0.77 ± 0.13	1.12 ± 0.20	0.81 ± 0.19	0.44 ± 0.1	0.43 ± 0.2
TRPV1_v4_	0.64 ± 0.11	0.61 ± 0.08	0.16 ± 0.01	0.40 ± 0.02	0.22 ± 0.1	0

B)			
TRPV1_v3_	(n= 6/44)	(n= 22/44)	(n=16/44)
Rel. Expr.	0	≤ 0.50	> 0.50
% of samples	13.6	50	36.4

C)			
TRPV1 5'UTR variants	HNPC (n=3/3)	GSC (n=2/4)	dGSC (n=4/4)
TRPV1_v0_	0.42 ± 0.02	0	0.23 ± 0.01
TRPV1_v1_	0.33 ± 0.02	0	0.27 ± 0.01
TRPV1_v3_	1.02 ± 0.13	0.25 ± 0.01	0.38 ± 0.02
TRPV1_v4_	0.22 ± 0.01	0	0.15 ± 0.01

A) TRPV1 5'UTR variants relative expression (Rel. Exp) levels in Normal Brain (NB), Normal Human Astrocytes (NHA) and in glioma biopsies from grade I to IV. B) TRPV1_v3_ expression levels in GBM samples, subdivided in three categories: no TRPV1_v3_ expression (Rel. Expr. = 0), low TRPV1_v3_ (Rel. Expr. ≤ 0.50) and high TRPV1_v3_ (Rel. Expr. > 0.50). TRPV1_v3_ relative expression levels are indicated. C) TRPV1 5'UTR variants relative expression (Rel. Exp) levels in human normal progenitor cells (HNPC), glioma stem-like cells (GSC) and dGSC (differentiated glioma stem-like cells). n: indicates the number of samples analyzed. A, B) n: indicates the number of TRPV1 positive samples respect to the total samples analyzed. A-C) Data are represented as mean of TRPV1 relative expression ± S.D. of three-separated RT/PCR analysis. Relative values were normalized to GAPDH expression level.

Taken together, these results revealed that TRPV1_v3_, which is the most stable TRPV1 5'UTR variants, is responsible for the TRPV1 expression in GBM and GSCs TRPV1^+^ samples, while the complete profile of TRPV1 variants expression was present in NB, NHA and HNPC samples, and with different expression levels in Grade I-III gliomas.

### TRPV1 expression and patient survival

**Figure 4:**
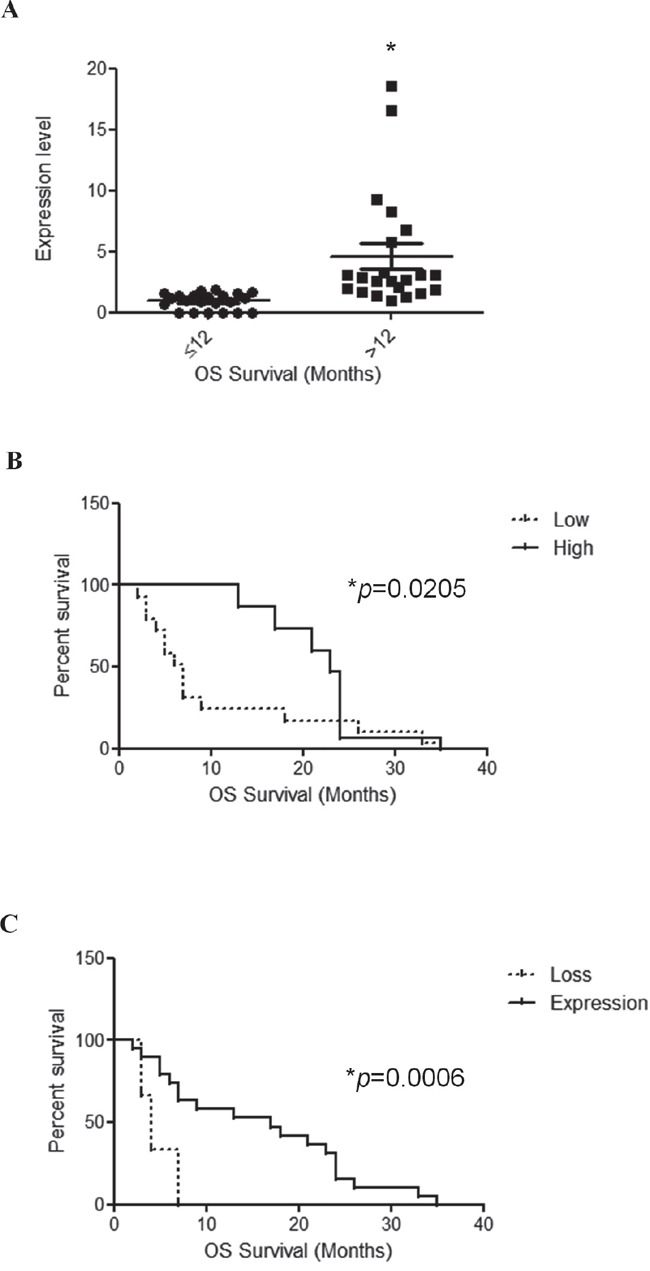
TRPV1_v3_ mRNA expression correlates with survival **A.** Distribution of TRPV1_v3_ mRNA expression in short survival (OS < 12 months) and long survival (OS > 12 months) GBM patients (n = 44 GBM specimens). Statistical analysis was performed by Unpaired t test at two-tailed (P < 0.05) value. **B.** Kaplan-Meier survival analysis for TRPV1_v3_ expressing glioma patients, illustrating an association between: A) survival and TRPV1_v3_ mRNA expression based on two categories, high (fold > 2.0) and low (fold < 2.0) TRPV1_v3_ levels. **C.** survival and TRPV1_v3_ mRNA expression based on two categories, TRPV1_v3_ mRNA loss vs expression (n = 44 samples). Associated log-rank test and Cox regression P-values were indicated.

TRPV1 mRNA expression was correlated with patients overall survival (OS). We first calculated the mean and median OS of GBM patients. We found that the mean OS was 14.4 months and the median OS was 11.0 months. Then, we evaluated the significance between patients survival (short OS survival < 12 months and long OS survival > 12 months) with the TRPV1_v3_ mRNA expression levels of all GBM patients (n = 44) by unpaired *t*-test. TRPV1_v3_ mRNA expression reached significance (p = 0.0009) for survival with short OS GBM patients, showing lower TRPV1_v3_ mRNA expression compared with long OS patients (Figure 4A). Moreover, on univariate analysis, survival curves were calculated according to the Kaplan–Meier method by evaluating TRPV1_v3_ mRNA expression of all patients. TRPV1_v3_ mRNA expression reached significance for survival (p = 0.0205) (Figure 4B and Table 4A). Similar results were obtained in a subgroup of GBM patients (n = 6) showing complete loss of TRPV1_v3_ mRNA expression, as respect to TRPV1 expressing GBM patients (n = 38) (p = 0.0006) (Figure 4B and Table 4B). Thus, low or absent of TRPV1_v3_ expression strongly correlates with short survival in GBM patients. Additionally we performed a multivariate analysis based on the Cox regression model to test the influence of TRPV1_v3_ mRNA expression on the survival of all patients or those with deleted TRPV1_v3_ expression. We found that the degree of TRPV1_v3_ mRNA expression retained its significance as an independent prognostic factor in all group of patients (P = 0.0008) and in the subgroup with deleted TRPV1_v3_ expression (P = 0.0011) (Figure 4C and Table 4B).

**Table 4:** Statistical analysis of TRPV1_v3_ in GBM

Table 4A: Univariate Kaplan-Meier survival analysis and TRPV1_v3_ mRNA expression (log-rank test)				
	All patients (n = 44) N° events (n = 38)		All patients (n = 44) N° events (n = 44)	
	Log-rank test	P-value	Log-rank test	P-value
TRPV1_v3_ (low/high)	5.367	0.0205		
TRPV1_v3_ (loss/expression)			11.90	0.0006

Table 4B: Multivariate Cox proportional hazard regression analysis of Kaplan-Meier survival analysis of TRPV1_v3_ mRNA expression in relation to survival rates	
	Relative risk (95% CI) All patients (n = 38/44)
TRPV1_v3_ (low/high)	2.25 (1,13-4.45) P = 0,0008
	**Relative risk (95% CI) All patients (n = 6/44)**
TRPV1_v3_( loss/expression)	17.1 (3.40-85.10) P = 0.0011

## DISCUSSION

Deregulation of gene expression is a hallmark of cancer cells. Acquiring or maintaining a specific profile of expressed proteins may enable the tumor cells to grow, migrate or survive to apoptotic stimuli. Modifications of mRNA stability and/or translation efficiency are increasingly reported in cancers [15, 26]. The UTRs flanking the coding region in mature mRNA regulate translation or mRNA stability through diverse mechanisms, representing an extra level of gene expression control [26-28]. An increasing number of reports highlight the effect of simple sequence elements or secondary and tertiary structures of the 5’ UTR in affecting the translation efficiencies of proto-oncogenes or tumor suppressing genes [15], neurotransmitter receptors [29] and ion channels [30]. Elements in the 5’ UTR can mediate translational regulation via the 5’-cap or the secondary structure, and stable 5’ UTR structures can interfere with translation by reducing the accessibility for the translational machinery and ribosomal scanning [31-33]. The TRPV1 channel, whose regulation was investigated in glioma, evidenced different expression patterns. Our results indicated that among the TRPV1 5'UTR variants, TRPV1_v3_ has the most structured and stable 5'UTR sequence, since its secondary 5'UTR structure shows the lowest free energy compared with the other 5'UTR variants. Moreover, experiments using Act D confirmed that TRPV1_v3_ in GBM is the most stable TRPV1 5'UTR transcript. Secondary structure and 5'UTR elements can function as major regulatory tool in 5'UTR and are prevalent among mRNAs poorly translated under normal conditions [34]. Our data suggest that TRPV1_v3_ is the only representative transcript expressed in TRPV1 positive GBM samples analyzed. Further computational prediction analysis revealed that all TRPV1 5'UTR variants contain a common translational sequence, named TOP, and TOP mRNAs remain sensitive to Rap treatment. In various cell lines, the degree of TOP inhibition by Rap varies, indicating that the effects of Rap are most likely cell type dependent [35-37]. Our results indicated that in GBM cell lines, treated with Rap, the TRPV1 protein levels decrease, suggesting that TRPV1 translation is sensitive to Rap. Since the pro-survival role of autophagy in tumors, including GBM [38], down-regulation of TRPV1 protein by a canonical autophagic inducer is interesting also in the view of a counterattack role of the TRPV1 channel in autophagy, through the induction of apoptosis in GBM cells [22]. Another regulatory element that was evidenced by computational analysis, is the GAIT element, that was found in 5’ UTR of TRPV1_v0,v3,v4_. GAIT element has been experimentally demonstrated to be sufficient for mRNA translational silencing, by IFN-γ, in human U937 monocytic cells [39]. Our results confirmed that INF-γ is able to reduce TRPV1 protein levels compared with untreated cells, suggesting that GAIT can be another 5'UTR element able to regulate TRPV1 translation. Altogether, these results evidenced the role of 5'UTR in TRPV1 translation and stability, suggesting novel potential mechanisms to regulate TRPV1 functions in human GBM cell lines. Our and other research data indicate that triggering of TRPV1 by synthetic or endogenous ligands activates apoptotic cell death pathway [22, 23]. Thus, it is conceivable that the reduced or lost expression of TRPV1_v3_, the only TRPV1 variant expressed in GBM, could represent a mechanism by which the GBM cells evade pro-apoptotic signals. Moreover, this hypothesis was supported by the analysis in grade I-III gliomas, where we found that TRPV1 variant expression levels were inversely correlated to the glioma grade. In addition our findings evidenced that, as in TRPV1^+^-GBM samples, GSCs maintain only TRPV1_v3_ transcript. Since different mechanisms mediate the resistance of GSCs to cytotoxic therapies and to pro-apoptotic signals [40-42], including down-regulation of TRPV1 [23], this data suggest that reduced TRPV1 expression in GSCs could be associated to their acquired resistance, as in GBM.

Furthermore, herein we showed that by inducing GSCs differentiation the TRPV1_v3_ expression increased, and the expression profile of dGSCs was similar to that found in low-grade glioma samples. Accordingly to our data, loss of TRPV1 was associated to proliferation of neural stem cell and neurogenesis reduction [23].

In support of this hypothesis, we found that the reduction of TRPV1_v3_ is accompanied by poor prognosis, which is also worsened by the complete loss of TRPV1_v3_ mRNA expression. In the same view, TRPV1 mRNA was found to be over-expressed in GBM tissues as respect to NB and TRPV1 expression was significantly higher (5-fold) in GBM patients with higher OS (more than 12 months) as respect to that showing lower OS (less than 12 months) [25]. No data on the expression of TRPV1 variants in tumors have been provided so far. The only data in cancer is referred to TRPV1 wild type. Thus, in hepatocellular carcinoma, TRPV1 expression correlates with better prognosis of patients [43], while in bladder cancer its reduction represents a prognostic negative biomarker [21].

Overall our results suggest that TRPV1_v3_ expression is a potential negative prognostic marker for patients with GBM. In fact, the reduction or null expression of TRPV1_v3_ transcript was associated with a short survival of all these patient groups. In a multivariate Cox proportional hazards regression model, TRPV1_v3_ expression reached significance as an independent prognostic factor for survival considering all patients and the subgroup characterized by loss of TRPV1_v3_ expression.

All together, these data suggested that TRPV1 could have a role in tumor aggressiveness, since loss of reduction of TRPV1 expression is associated with a more proliferative tumor, as also reported for others cancer cell types [20, 22, 43].

In conclusion our results, showing that the TRPV1_v3_ has the lowest free energy in its secondary 5'UTR structure and the highest 5'UTR sequence stability, suggest that it may represent a potential prognostic marker for patients with GBM. As an independent prognostic factor for poor disease outcome, TRPV1_v3_ may be useful to improve appropriate selection of post-operative follow-up protocol for individual patients. Further studies with a more representative number of GBM samples, are required to completely assess the role of TRPV1 5'UTR identified in GBM patients.

## MATERIALS AND METHODS

### Software for mRNA structure analysis

RegRNA 2.0 was used for identifying functional RNA motifs in 5’ UTR TRPV1 RNA sequence [44]. MView reformats the results of a sequence database search (BLAST, FASTA, etc) or a multiple alignment (MSF, PIR, CLUSTAL, etc) and was used for TRPV1 variant transcripts comparative analysis [45]. The structure and the free energy (ΔGs) of the TRPV1 variant transcripts were calculated using RNAfold [46].

### Cell cultures and human bioptic samples

U87 and U251 human glioma cell lines were obtained from the American Type Culture Collection (LGC Promochem, Teddington, UK). Glioma cell lines were growth in Dulbecco's Modified Eagle's medium supplemented with 10% fetal bovine serum, 2 mmol/L-glutamine, 100 IU/mL penicillin, 100 μg streptomycin at 37°C, 5% CO_2_ and 95% of humidity.

Formalin-fixed paraffin-embedded glioma tissues from human tumor biopsies surgically removed from patients who gave informed consent to the study (n = 84), were kindly provided by Prof. Felice Giangaspero (I.N.M., Neuromed, Pozzilli, Isernia, Italy) as previously described [47]. GSC lines previously characterized [42] were cultured in a serum free medium supplemented with 20 ng/ml of epidermal growth factor (EGF) and 10 ng/ml of basic fibroblast growth factor (bFGF). For differentiation, GSC lines were grown for 15 days, in medium supplemented with 5% fetal bovine serum (FBS). HNPC were purchased from Cambrex, (UK) and cultured folllowing manufacturing instruction.

### Compounds

Rap, Act D and IFN-γ were purchased from Sigma-Aldrich (Bristol, UK), and aliquots of 25 mM were prepared according to manufacturing instruction.

### RT/PCR analysis

Total RNA (RNAt), from cell lines, was extracted using the RNeasy Mini kit (Qiagen, Milan, IT). RNAt from GBM biopsies was extracted with RNeasy FFPE kit (Qiagen, Milan, IT), as previously described [44]. Total RNA from NHA and NB were purchased (Clonetech, USA). Complementary DNA (cDNA) was synthetized from 1 μg of RNAt using the high-capacity cDNA archive kit (Life Technology, Milan, IT), in a total volume of 25 μl. Real Time Quantitative PCR (qRT-PCR), for TRPV1 variants was performed using specific primers (Supplementary Table 3) and human GAPDH RT^2^ qPCR Primer assay (Qiagen) were used to normalize qRT-PCR data. The following thermal cycling conditions: 3’ at 95°C; 35 cycles of 10” at 95°C, 20” at 60°C, 20” at 72°C; and finally 50” at 72°C, using iQ5 Thermal Cycler (Bio-Rad, Hercules, CA). Melting curve was performed to verify primers specificity. One μl of cDNA was used in a final volume of 25 μl, with 2X RT^2^ SYBR^®^ Green qPCR Master mixes (Qiagen) and each sample was amplified in triplicate. Measurement of GAPDH transcript level was used to normalize TRPV1 variants levels. TRPV1 variants levels were calculated by the 2^−ΔΔCt^ method and expressed as relative fold compared with the corresponding control.

### RNA stability assay

Glioma cell lines were treated up to 12 h with Act D (10 μ g/ml) and RNAt was isolated from the cell pellet using RNeasy mini kit, and eluted in 50 μl of H_2_O. The same volume of RNAt samples were run in 1.5% agarose gel electrophoresis, then gel was visualized by UV trans-illuminator and the image of the gel was acquired by Chemi-Doc (BioRad). Real time-PCR with the specific TRPV1 5'UTR variants primers was performed. For each sample, the amount of TRPV1 transcripts was evaluated relative to 1 μg of RNAt, by Real-Time PCR. Briefly, first-strand cDNA was synthetized using 1 μg of RNAt and 10 ng of cDNA, for each sample, was amplified with specific TRPV1 variant primers. Experiments were performed in triplicate.

### Western blot analysis

Glioma cell lines were lysed as described previously [44]. Twenty micrograms of the lysates were separated on a SDS polyacrylamide gel, transferred onto Hybond-C extra membranes (GE Healthcare), blocked with 5% low-fat dry milk in PBS-Tween 20, immunoblotted with mouse monoclonal anti-TRPV1 antibody (Ab) (1:1000, Sigma Aldrich), and mouse monoclonal anti-glyceraldehydes-3-phosphate dehydrogenase (GAPDH) Abs overnight and then incubated with HRP-conjugated Ab (1:2000, Cell Signaling) for 1 h. Immunostaining was revealed using an enhanced chemiluminescence Western blotting analysis system (GE Healthcare). Densitometric analyses were performed by ChemiDoc using the Quantity One software (Bio-Rad).

### TOP analysis

Glioma cell lines were treated for 24 h with Rap (10 μM), then cell pellets was lysed for western blot analysis.

### GAIT analysis

GAIT regulation of TRPV1 variants translation was determined, by treating glioma cell lines with IFN-γ (100 μM) for 24 h. Western blot analysis were performed to evaluate TRPV1 protein levels.

### Statistical analysis

The statistical significance of Ct variation levels was determined by Student's *t*-test. Statistical analysis was performed using the Kruskal–Wallis test for comparison of TRPV1 mRNA expression groups. *P*-value ≤0.05 was considered significant. Patient follow-up was evaluated as the number of months from the date of the diagnostic surgical procedure to that of last visit or death. Survival time was defined as the period between the time of diagnosis and the time of death.

Statistical analysis was performed using GraphPad Prism 9.0 software (GraphPad software, San Diego, CA, USA). Univariate survival analysis was performed according to the Kaplan–Meier method, and differences in the survival curves were assessed with the log-rank test. Multivariate survival analysis was performed using Cox's proportional regression analysis and the relative risk was calculated with 95% confidence interval (CI). A P-value ≤0.05 was considered statistically significant.

## SUPPLEMENTARY MATERIALS TABLES


