# Supplementary material for: Post-transcriptional regulation of 5'-untranslated regions of human Transient Receptor Potential Vanilloid type-1 (TRPV-1) channels: role in the survival of glioma patients
Source: Oncotarget. 2016 Nov 5;7(49):81541–54. doi: 10.18632/oncotarget.13132 (PMC5348411; doi:10.18632/oncotarget.13132)
Supplement: Supplementary file 1 [file oncotarget-07-81541-s001.pdf]

Post-transcriptional regulation of 5'-untranslated regions of human Transient Receptor Potential Vanilloid type-1 (TRPV-1) channels: role in the survival of glioma patients

SUPPLEMENTARY TABLES

Supplementary Table S1: Primer sequences for TRPV1 5'UTR variants amplification

TRPV1 splice variant	Forward primer	Reverse primer
TRPV1$_{v0}$	5'-TGCCTGCGTCTAGCTGGTT-3'	5'-CTGTGCTGCTCCATTTCTTCAT-3'
TRPV1$_{v1}$	5'-CACAATCCCGGCTGCTTCCT-3'	5'-CGGCGTTGCCTTTTCCT-3'
TRPV1$_{v3}$	5'-TCACCGAGATCCTCCCTGAA-3'	5'-CCCCACACAGAACAAGGAAAA-3'
TRPV1$_{v4}$	5'-CACAATCCCGGCTGCTTCCT-3'	5'-CGGCGTTGCCTTTTCCA-3'

Supplementary Table S2: 5'UTR length indicated as number of nucleotides (nt), for each TRPV1 variant

TRPV1 5'UTR variant	5' UTR lenght (nt)
V_0	158
V_1	275
V_3	527
V_4	201

Supplementary Table S3: Clinicopathological characteristics of 44 GBM patients analysed for TRPV1 5'UTR variant mRNA expression

Patient characteristics	
n° of samples	44
Sex	
Male (%)	24
Female (%)	20
Age (Years)	
< 45	34
>45	50
